# Closing the Gap: Refining Vaccine Forecasting and Resupply Calculations in Mozambique Through Advanced Routine Data Analysis

**DOI:** 10.3390/vaccines14020118

**Published:** 2026-01-27

**Authors:** Wendy Prosser, Laila A. Akhlaghi, Santos Sipaneque, Tito Rodrigues

**Affiliations:** 1 JSI Research and Training Institute, 2733 Crystal Drive, Arlington, VA 22202, USA; 2 Ministry of Health Mozambique, Avenida Eduardo Mondlane, na cidade de Maputo No. 1008, Maputo P.O. Box 264, Mozambique

**Keywords:** sustainable vaccine supply and procurement, forecasting, wastage rate, immunization supply chain

## Abstract

Background: Vaccines save lives, but only if they are available at health facilities for administration. Stockouts occur for various reasons, including inaccurate forecasting and resupply calculations. Population-based forecasts are typically used for immunization programs, yet they are often based on inaccurate population estimates. This retrospective study analyzed available routine facility-level data from two districts in Mozambique to provide insights for improved supply chain management, including resupply decisions, at the facility level. Methodology: Data from August to October 2023 were collected and analyzed for wastage rate, session cohort, and forecast accuracy. Results: The results show that district-level wastage rates are nominally different from globally acceptable standards, while being significantly different at the facility level. Analysis also showed divergence of vaccination doses provided to a session cohort during the month-long periods that appear to be correlated with periods of stockout. Using population-based forecasting for resupply methodology consistently undersupplied facilities by 20% (ranging from 5 to 41% across 16 facilities), while using the number of doses of administered BCG as a proxy for the population oversupplied by 12% (ranging from 1% underforecast and 28% overforecast), with tighter variance. Conclusions: Despite limitations due to the availability and quality of data, the results suggest an opportunity to shift from a traditional population-based approach to forecasting and resupply decisions, leveraging existing data systems, applying tailored wastage rates, and adjusting inventory management policies to ensure vaccine availability.

## 1. Introduction

Immunization is one of the most cost-effective and beneficial health interventions to improve health and reduce vaccine-preventable diseases, particularly in young children [1]. Since the establishment of the Essential Program on Immunization (EPI) five decades ago, an estimated 154 million lives have been saved through vaccines [2]. Continued global commitment to immunization is described in the Immunization Agenda 2030, setting a vision wherein vaccines are available for all to provide protection from disease [3]. While impressive increases in vaccine coverage have been made in the past decade worldwide, research has also identified a few standard causes of children being unvaccinated or under-vaccinated, including remoteness from and difficult access to health services, low education level or socioeconomic status of the mother or caregiver, low maternal use of health services, and health system factors such as vaccine stockouts at the health facility level [4,5].

Having a reliable supply of vaccines available at the service delivery point is critical to ensuring timely vaccine coverage. One study in South Africa found that 62% of missed vaccinations were due to stockouts at the facility level [6]. Additional studies have also shown the negative impacts of vaccine stockouts on coverage rates and trust in the health system [7,8]. As a 2017 global analysis of stockouts in African countries shows, the frequency and degree of stockouts vary between and even within countries, with an estimated 50% of these countries reporting stockouts of at least one vaccine for at least one month at the national and district levels [9], as well as notable stockouts at the facility level [10].

One reason noted for stockouts is inadequate forecasting and stock management, accounting for 18% of national and sub-national stockouts in one global analysis conducted using data from 2011 to 2015 [11]. The World Health Organization (WHO) provides guidance on three methods to forecast vaccine needs: using the target population, previous consumption, or the size of the immunization sessions [12]. Most low- and middle-income countries typically use target population estimates as the main national-level forecasting methodology, which can result in forecasting error, as it is well documented that target population estimates are often inaccurate due to internal migration, unregistered births, and growth rate misestimation, particularly at lower local levels [13,14,15].

The population-based forecasting method begins with population figures and applies multiplication of various assumptions to reach the total number of vaccines needed. The population figures are determined by the demographic and age groups that are to receive immunizations, such as pregnant women, girls aged 9–14, infants, and surviving infants. Additional assumed factors include growth factors, EPI schedule, coverage rates per scheduled dose, and wastage rates. A 25% buffer stock is added to mitigate unpredictable variability in supply and demand.

Global standard vaccine wastage rates are typically used as the standard to calculate vaccine needs and are determined by characteristics of the vaccine (i.e., vial size; lyophilized versus liquid) [16]. These global standards may not reflect the nuances of national or regional immunization services. Planning tools, such as the WHO Wastage Rate Calculator [17], are available; however, they focus on national-level estimates and, thus, miss the granularity of sub-national-level differences. Furthermore, studies have shown the binomial nature of wastage rates and how rates can be calculated for facility-level open-vial wastage, yet this relationship depends on the session size, which is not readily available through reporting systems [18]. These standards, when applied as assumptions across the aggregate dataset, introduce errors and miscalculations for national forecasts. These errors and miscalculations are compounded as vaccines move through the supply chain, impacting resupply calculations for facility-level vaccine needs.

The challenge of inaccurate forecasts and target populations is particularly salient in settings characterized by infrequent censuses, demographic complexity, and resource constraints, such as in Mozambique. Mozambique has traditionally used the census population from the National Statistics Institute (INE in Portuguese), adjusted by estimated annual growth, as the basis of its forecast. Mozambique has recently introduced different methodologies for the national vaccine forecast [19]; however, this has yet to be fully adopted and applied at the sub-national or facility levels. Vaccine distribution typically follows a four-tier system from national to provincial, to district, and to health facilities, with some slight variations between provinces, using the standard INE population for the forecasts with wastage and buffer added. Anecdotally, health facilities obtain emergency top-ups between scheduled distribution cycles in the event of stockouts, but these top-ups are often not reflected in the information management system. Stockouts at the facility level are a common event [20,21]. While many factors influence stock availability at the facility level, using the target population to estimate vaccine needs is likely a contributing factor.

In this context, this study analyzed available routine facility-level data in Mozambique to inform and provide insights for improved supply chain management, including resupply decisions, at the facility level.

## 2. Methodology

### 2.1. Study Design

This retrospective study used purposeful sampling to select two districts, Nhamatanda and Gorongosa, in Sofala Province, Mozambique, and included all health facilities in each district (22 and 20, respectively, at the time). These two districts were specifically selected for inclusion in this study due to their notable similarities in key socioeconomic indicators and similar healthcare infrastructure. According to INE 2024 estimates, both districts have similar population sizes (349,422 in Nhamatanda and 218,788 in Gorongosa) and comparable urban/rural population distribution pattern ratios. A separate analysis for forecast accuracy was conducted in Gorongosa based on available LMIS data for 12 months.

### 2.2. Data Collection

Routine administrative data on service delivery and supply chain reporting were collected for three months (August to October 2023) from the government’s Health Management Information System (HMIS) and the immunization Logistics Management Information System (LMIS). Additional facility-level data were collected for the same period, using stock cards of all vaccines, monthly facility reports on immunization activities, and daily summary sheets. Daily summary sheets track vaccines administered and vials opened each day and are used to aggregate data for the monthly facility reports. Target population estimates for each district were also obtained from the INE. Table 1 provides details of the data sources and data points collected.

A data collection plan was developed. Four research assistants were trained on the content and the data collection tool. This was followed by a pre-testing of the tool in two facilities. Adjustments of the tool were made based on the results of pre-testing. The facilities were informed about the study, and dates were scheduled for the visit. Each data collection team reported to a supervisor daily.

Data collection was conducted using SurveyCTO. Data were collected from the stock cards. Photos were taken of daily summary sheets, and data were input into SurveyCTO at a later time. The provincial team extracted LMIS and HMIS data for all facilities in the two provinces. Data were collected on all child vaccines and the EPI schedule based on the session cohort; HPV and tetanus were omitted because they target different populations (Table 2 and Table 3).

### 2.3. Data Management and Analysis

Data were reviewed and cleaned before the analysis, including the elimination of facilities that did not have complete datasets for the period or did not provide certain vaccines. For easy management, data were captured and analyzed in Excel, and the results were presented in tables and graphs generated using both Excel and Tableau. Based on available data, the analysis considered three primary outcomes: wastage rate, session cohort, and forecast accuracy themes.

The first outcome was calculated wastage rates for each vaccine per facility using existing data in Gorongosa District only. Wastage was defined as the number of vaccine doses that were consumed or used but not administered to a person.

The reported consumption data from the LMIS were compared with doses administered as reported through the HMIS. What was consumed but not reported as an administered dose was calculated to be wastage, as in the following calculation:

$$\mathrm{Wastage}\;\mathrm{rate}\;=\;\frac{\left(consumption\;(LMIS)-\;doses\;administered\;(HMIS)\right)}{consumption\;(LMIS)}$$

This calculation was carried out for each vaccine, month, and facility. Notably, the analysis did not include the HMIS monthly reported open- and closed-vial wastage as the completeness and quality of this data is consistently low.

The analysis also calculated the district-level wastage rate by averaging the various facility-level and monthly wastage rates into one representative district wastage rate. The calculated wastage rates by vaccine were compared with global acceptable standards.

The analysis did not distinguish between closed-vial or open-vial wastage due to the lack of nuanced data. Closed-vial wastage is often due to expiry, vial breakage, or exposure to extreme temperatures (heat or freezing), while open-vial wastage is often an inevitable part of immunization programs using multi-dose vials.

The second outcome considered the extent of the differences, or divergence in number of vaccines administered as expected per the session cohort based on the EPI schedule. The session cohort was defined by the age groups expected to receive the same number of vaccines per schedule and visit, aggregated by month. For example, based on the EPI schedule in Mozambique, a two-month-old should receive one dose each of DPT, OPV, PCV, and rotavirus during the same visit. For this analysis, the percentage difference in total doses administered of each vaccine in the session cohort was compared with the maximum doses given per cohort/schedule by month. The maximum, or highest volume of any of the scheduled doses of the session cohort, was used as the reference point for calculating the variance of all other vaccines within the cohort. Each vaccine within the cohort would have its own percent difference from the maximum doses administered in the cohort, with the reference always being 0%. Pearson’s correlation coefficients (*r*) were calculated to determine the strength and direction of the divergence from the maximum number of vaccines administered within each session cohort and vaccine stockouts [22]. The calculation is described as follows:

$${Divergence}_{vaccine\;in\;cohort}=\frac{{Doses\;administered}_{max\;in\;cohort}-{Doses\;administered}_{vaccine\;in\;cohort}}{{Doses\;administered}_{max\;in\;cohort}}$$

In the absence of a gold-standard comparison, this serves as a practical proxy for an established benchmark. Divergence from the maximum scheduled doses in the session cohort implied missed opportunities to vaccinate and was correlated with reported stockouts at the end of each month.

Finally, the third outcome analyzed forecast accuracy in Gorongosa District for 12 months, comparing two methodologies for resupply calculations at the facility level: the traditional INE target population estimates, as determined by census data and extrapolated for subsequent years; and the reported number of BCG doses administered the previous year as a proxy for the target population (infants), justified by a high BCG coverage rate (84%) and a relatively high adherence to first antenatal care visits (87%) and facility-level births (65%) [23]. Forecast accuracy was estimated by calculating the difference between the number of doses administered and those projected by the two different estimates, divided by the selected forecast. The calculation was as follows:

$${Forecast\;error}_{variation}=\;\frac{Forecast\;-\;Actual\;}{Actual}$$

For example, if 110 doses were administered, and the INE-based estimates forecasted for 100 doses, the result is −10% (or 10% underforecasted). If the BCG-based estimates forecasted for 120 doses to be administered, the result for BCG is 8.3% or (8.3% overforecasted). Values above 0% would reflect an overforecast, with 0% as accurate, and below 0% as an underforecast. Forecast error is usually calculated with the numerator as “Actual - Forecast”; however, a variation was used in this situation to ease interpretability, with negative values representing underforecasts and positive values representing overforecasts.

Secondary outcomes looked at the completeness of reports, the frequency of stockouts, and the number of children immunized to provide insight into the study setting and context.

Data collection and analysis procedures were coordinated and conducted with the immunization program of the Ministry of Health and the Provincial Directorate of Health.

## 3. Results

Data from the HMIS were collected for all facilities in the two districts from August to October 2023. LMIS data were collected only for Gorongosa District, as the LMIS had not yet been deployed in Nhamatanda. The period covered was from July to October to capture the beginning stock balance for August as reported at the end of July. Daily summary sheets were collected from 30 facilities, but the lack of data completeness prevented their inclusion in this analysis. Stock data were collected from stock cards at the facility level, but they were also not included in the analysis because they were often incomplete. For further analysis of forecast accuracy in Gorongosa, HMIS data from January to December 2023 were included in the data collection. For Gorongosa, 16 out of 17 facilities had datasets for all usable sources for the duration of three months. Data collection was conducted in November and December 2023 (Table 4).

We reviewed the LMIS and HMIS data for the 16 facilities that reported to both systems for the three months. The calculated consumption data were very closely aligned with the reported consumption data in the LMIS. The results revealed that of the 480 instances (facilities by vaccines), there was a 74% match between doses opened in the HMIS and doses consumed in the LMIS; 6% were not matching; and 10% had one field left empty (Table 5).

Additionally, using data only available in Gorongosa, using zero stock at the end of a month to measure a stockout, an average of 32% of the vaccines and 27% of the facilities experienced a stockout, varying across vaccines between 13% and 41% (1st to 3rd quartiles), and varying across facilities between 11% and 44% (1st to 3rd quartiles). The duration of the stockouts is unknown. There were no reported national-level stockouts during the study period.

**Figure 1:**
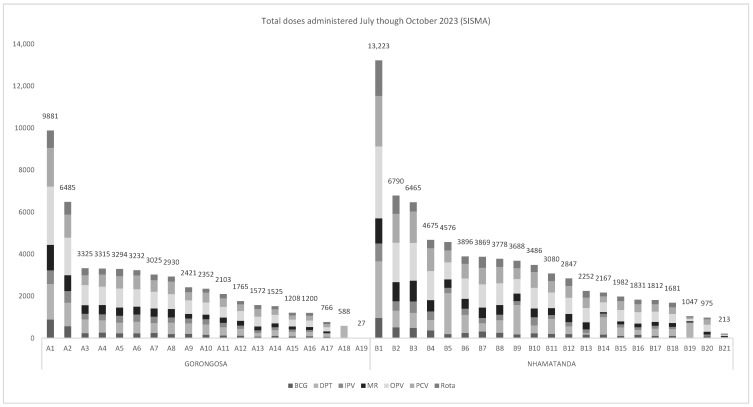
Total number of vaccines administered by facility, July–October 2023.

Figure 1 shows the total number of vaccines administered by facility, as reported in the HMIS, reflecting the variation in facility size and target populations.

### 3.1. Wastage Rate

**Figure 2:**
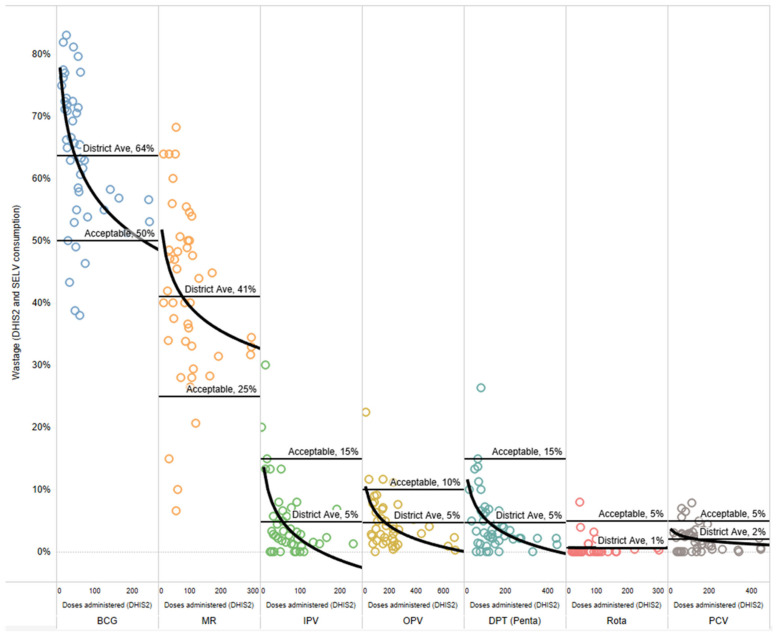
Results of wastage rates compared with doses administered as a proxy for session size, with binomial trend lines, district averages, and global standard acceptable wastage rates.

Wastage rate analysis is shown in Figure 2. The results show that the average wastage rates (calculated either from site wastage rates or from the total doses administered and consumed) at the district level according to data from the facilities are significantly different for all vaccines, with BCG and MR having a higher calculated wastage rate and the rest showing lower calculated wastage rates. For BCG and MR, the acceptable wastage rates are 50% and 25%; however, the calculated wastage rates at the district level were 64% and 41%.

Particularly compelling insights emerged from reviewing the facility-level wastage rates, which indicated a large range of wastage rates across vaccines. For example, particularly notable is the high variability for the two lyophilized vaccines—BCG and MR—with wastage rates ranging from 38% to 82% and from 7% to 68%, respectively. Each dot represents a different facility and month, reflecting the variability, local realities, and/or quality of the reported data.

Session size—specifically, the number of children attending a facility for vaccine services—is a key factor influencing wastage rates, especially for lyophilized vaccines. The analysis compares wastage rates to the number of doses of that vaccine administered at the facility per month as a proxy for session cohort since the session size is not a readily available indicator. Logically, facilities with a lower volume of doses administered in a month (likely linked to smaller facilities or population targets) will result in higher wastage rates, particularly for BCG and MR. The outcomes are similar to those previously identified in studies that have demonstrated this relationship between the birth cohort of the catchment area and the session frequency. Wastage rates in the negative indicate a gain in the system and are likely a result of data quality issues.

### 3.2. Completeness of Recommended Vaccine Administration per Age Group, Session Cohort, and Schedule

Inconsistencies in doses administered by session cohort were identified in all facilities. Despite the co-administration schedule, the analysis shows a divergence in the quantity of vaccine doses administered and scheduled for simultaneous delivery per session cohort. If data was reported accurately and all doses recommended per age group were given, the total doses administered per session cohort would be the same quantity. One example of this analysis of a single facility for the two-month-old cohort in August indicates that 103 children were vaccinated with their first DPT dose, 88 for OPV- 1, 92 for PCV- 1, and 101 for RV- 1.

Although the reason for the divergence in doses administered in a cohort is unknown, the analysis considered stockouts as one plausible reason; analysis shows that the higher the divergence, the more likely there was a stockout. Pearson’s correlation was used to test the strength of correlation between stockouts, using binary value of reporting any quantity of stock, or zero stocks at the end of the month, and the average calculated divergence from maximum doses administered in the session cohort, across all recommended scheduled uses for the vaccine.

**Figure 3:**
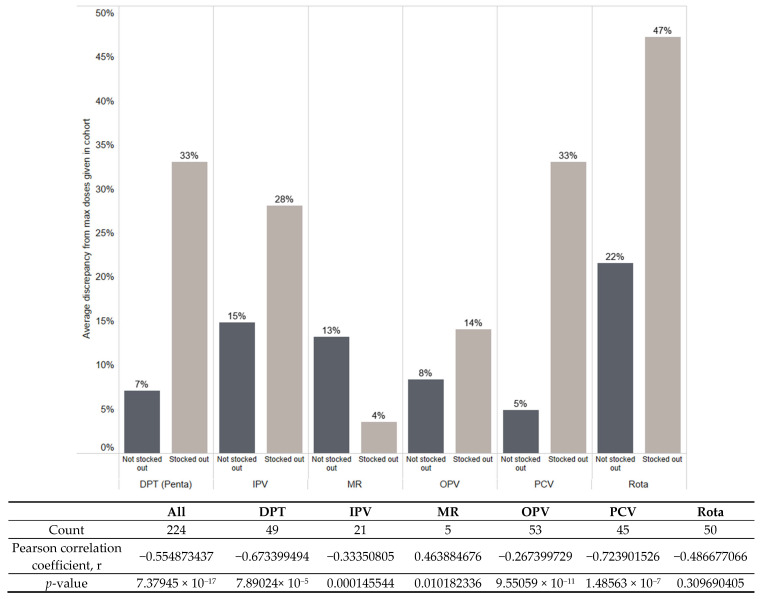
Average divergence from maximum doses per session cohort with reported stockouts at the end of the month, with count, Pearson’s correlation and *p*-values.

The results show that the extent of reported stockouts in a facility appears to be correlated with an increased divergence from the maximum scheduled doses in a session cohort. The strongest correlations appear to be with PCV and DPT, and the results for OPV are the least correlated (Figure 3). Since the number of instances for IPV and MR were 21 and 5, and the Pearson correlation can be applied to *n* > 30, our sample was not large enough to be conclusive for these two vaccines.

### 3.3. Forecast Accuracy

**Figure 4:**
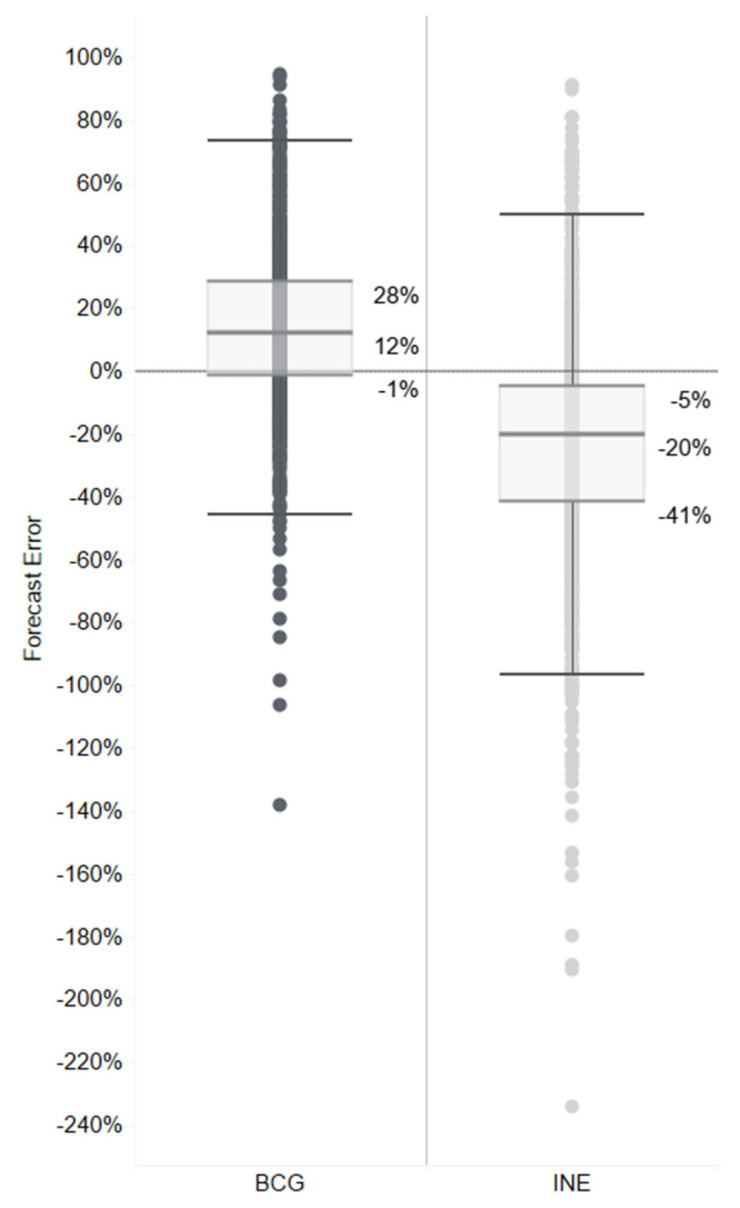
Range and quartiles of forecast error percent averages for doses administered compared with INE- and BCG-based forecasts across all vaccines and facilities. Negative errors indicate underforecast; positive errors indicate overforecast. Values exclude buffer stock and emergency transfers. Each dot represents a forecast error calculated per facility, vaccine, and month.

The results of the forecast accuracy analysis in Gorongosa District show that the BCG-based forecast provided an average of 12% more doses than used for all months, facilities, and vaccines (with half of the observations between 1% underforecast and 28% overforecast—a spread of 29%); and the INE-based forecasts provided an average of 20% fewer doses than used for all months, facilities, and vaccines (with half of the observations between 5 and 41% underforecasted—a spread of 36%) (Figure 4).

It is important to note that resupply decisions are not solely based on the projected number of doses to be administered. An allowance covers open-vial wastage; buffer stock is added to accommodate distribution disruptions and inaccurate forecasts; and the final order takes into consideration what is in stock at the time of the order. Since wastage rates and buffer stocks are determined as a proportion of the demand forecast, this additional allowance was not part of the analysis of the forecast for the demand for doses to be administered.

Additional data is available in the Supplemental Files, showing that at the facility level, fewer doses were forecasted using INE-based estimates (between 4% and 59% less) than were actually administered. On the other hand, with the BCG-based forecasts, on average, every facility would have overforecasted (4–24%). Similarly, the results viewed by vaccine also show underforecasts on average with INE-based population figures (between 10 and 42% less), with a range of accurate to overforecasts with BCG-based forecasts (0% to 21% more).

## 4. Discussion

Ensuring reliable vaccine availability at the health facility level is a requirement for the success of any immunization program. While numerous factors influence vaccines’ availability and use—including the accuracy of the national-level forecast, distribution logistics, cold chain functionality, and demand volatility—this study focuses on a single, pivotal aspect: the accuracy of resupply calculations and its possible connection to missed opportunities to vaccinate. We analyzed how optimizing these resupply calculations through more accurate wastage rates and tailored forecasts could improve planning to meet the actual demand for vaccines at the facility level and reduce the chances of missed doses. This analysis identified several aspects that could be further tested to improve the forecasting and resupply process, as well as priorities and management approaches for service delivery, particularly those related to data collection.

It has been noted that national-level forecasting is one of the weakest aspects in public health supply chains [24]. Inaccuracies in national-level forecasts can create inefficiencies and disruptions in the supply chain, inequitable distribution of vaccines, loss of productivity due to managing the disruptions, and financial waste for vaccine procurement. The results of inaccurate national-level forecasts can be exacerbated throughout the levels of the supply chain, resulting in stockouts at the facility level—increasing the risk of unvaccinated children and higher disease burden—or overstock, leading to higher wastages and costs. More accurate forecasts and resupply decisions can lead to a more efficient and resilient vaccine supply chain, mitigating the risks of both stockouts and costly oversupply.

This analysis demonstrates how using INE estimates of target populations for re-supply decisions consistently undersupplies the facilities in these two districts in Mozambique as compared with the true vaccine need based on the actual consumption, which should incorporate demand for doses administered and wastage. Our analysis shows that additional children were still vaccinated, even if facilities were undersupplied, which was likely mitigated by buffer stock, a lower wastage rate than was planned for, and presumably emergency top-ups or inter-facility sharing (not routinely documented). Supply chain best practices suggest that buffer stock should not be considered part of the forecastable demand, should consider resupply periods, and is meant to mitigate variability in both demand and supply (e.g., lead time). However, this analysis shows that for the immunization program in Mozambique, buffer stock is likely being used to address the demand resulting from underforecasts. This results in constraints and other workarounds to ensure vaccine availability when the buffer stock is not properly calculated to address an uptake in demand or delays in supply delivery and is used to address underforecasting. More accurate forecasts would reduce the reliance on and volume of buffer stock and could contribute to a higher number of children being vaccinated, with fewer stockouts and workarounds.

Using BCG as a proxy target population resulted in improved forecast accuracy, with some possibility of overstock. This may be a situation unique to this specific district, or even to Mozambique, due to relatively high facility-based births and high adherence to BCG for newborns. However, these results do make the case for using alternative forecasting methodologies to average out biases and errors, provide complementary information, leverage diverse data types, and improve the accuracy of the final forecast.

The other aspect of an accurate forecast is having accurate wastage rates. Quite logically, facilities that serve smaller populations had higher wastage rates, particularly for BCG as a 20-dose vial and MR as a 10-dose vial. This commonly occurs in immunization programs, and resupply quantities to these facilities should be adjusted accordingly. The globally acceptable standards for wastage rates have been the common guidance since the establishment of immunization programs, when the requisite data were not available for calculating accurate wastage rates. The global standards served a purpose, but with mature data information systems and modern analytics now available, immunization programs can more accurately calculate wastage rates for a tailored benchmark that reflects the reality of different health facilities and their communities [25].

An additional area for improvement is a better understanding of losses and adjustments. The logistics system in Mozambique, like in many countries, does not easily allow for reporting of losses and adjustments to record closed-vial wastage in the system. If losses and adjustments were part of reporting forms and designed to be easily used by staff, the closed-vial wastage rate could be calculated separately from open-vial wastage. With that insight, both open- and closed-vial wastages could be easily identified and addressed with different interventions.

The analysis related to session cohorts and missed opportunities identifies some gaps in service delivery through the divergence in the administration of vaccines in a session cohort. There are many reasons why a child might not receive a vaccine according to the schedule, such as failure to screen for eligibility, vaccine hesitancy, or stockouts, as corroborated by our results. Analyzing the data from a monthly aggregation to compare the vaccines that should be received by session cohort provides insight into the possible relationship between missed doses and stockouts. While the analysis does not provide a definitive explanation for the divergence, in the absence of daily reports or true session size, this methodology can serve as a diagnostic tool that guides supervisors in identifying and addressing the underlying factors affecting performance and areas for follow-up with healthcare providers.

This analysis demonstrates that while existing data and information management systems are available, their full potential is not being realized to address modern challenges in managing immunization programs and supply chains. This reflects the growing interest of governments and donors in investing in digital health and information systems, as well as the known challenges as these information systems are introduced [26]. The study’s results show that data completeness presented a challenge, particularly with the daily summary sheets and stock cards and their use in verifying LMIS and HMIS data. However, the analysis used data from the HMIS and LMIS, demonstrating that monitoring of immunization services can be achieved with these sources. The results showed a relatively high match between the LMIS and HMIS data reporting doses opened, indicating decent data quality yet with room for continuous improvement. Practical experiences show that consistently using and reviewing data will lead to quality improvements, as routine analysis helps to identify and correct errors, inconsistencies, and gaps [27,28]. The absence of robust feedback loops for facility-level monthly reports may contribute to underreporting. When health workers lack visibility regarding how their submitted data are used for decision-making, resource allocation, and stock monitoring, the perceived value of accurate and timely reporting diminishes, thereby weakening the incentive for consistent and thorough documentation.

This unique analysis challenges traditional population-based approaches to vaccine program management that have been the norm for decades by demonstrating that existing data, when analyzed with innovative methods, can provide nuanced and actionable insights. We have demonstrated the importance of reviewing each part of the forecast equation that determines how much product is stocked—the demand (e.g., accurate population estimates and appropriate forecasting methodologies), tailored wastage rates, and inventory policies (e.g., buffer stock)—and fine-tuning as data allow. This approach successfully identifies service delivery gaps (e.g., missed opportunities for vaccination), identifies possible errors in quality data reporting, and provides insight into the link between session cohorts and doses administered. This analysis makes the case for strengthening information systems at all levels of immunization programs and supporting more mature programs by using existing data sources in different and unique ways. These findings suggest that further research with a larger volume of data and across multiple provinces and countries would be beneficial to validate the results and revise standard methods of data use for improved insights.

## 5. Limitations

There are several important limitations to consider when interpreting our findings. The data were inconsistent and incomplete across data sources. Our analysis could not determine any distinction between open- versus closed-vial wastage, which is an important distinction for vaccine management, and while data quality issues were noted, it was not possible to determine the level of data quality errors that could influence the analysis. Additionally, no data were available on emergency top-ups of stock between distribution cycles, emergency orders, or inter-facility transfers, which creates a challenge in determining true, real-time stock levels at the facilities and may bias calculated wastage, underestimation of supply, and risks of stockouts. While data quality and completeness were noted to be insufficient, there are no complementary LMIS/HMIS assessments or verification protocols. Because of the study design, direct causation could not be determined between stockouts and missed opportunities to vaccinate, recognizing multiple influencers, including hesitancy, demand, and service delivery aspects. The sample in this analysis was sourced from only two districts in Mozambique and may not be generalizable to other areas or countries. The three-month period may not capture seasonal changes in vaccine demand. Future implementation research and quality improvement approaches should be undertaken to obtain a broader dataset for further analysis.

Notwithstanding these limitations, we believe that the current findings demonstrate the potential for a new data-driven framework to enhance supply chain management, thereby justifying subsequent exploration and validation.

## 6. Conclusions

Ultimately, this work illustrates a path forward for immunization programs to leverage their maturing data systems with innovative data analysis using existing LMIS and HMIS sources to move beyond standard approaches to supply chain management. This approach could be a step forward in using existing information for more accurate forecasting and resupply calculations, which is important for ensuring reliable vaccine availability and sustainable vaccine supply and procurement, as well as contributing to system efficiency. While more research is needed to validate any standardization of these methods, this analysis is the first step to demonstrating new approaches to using data to improve vaccine availability and contribute to system efficiency.

## Figures and Tables

**Table 1:** Data sources and data points.

Source	Data Point
Stock card, health facility	Opening balance Vaccines received Doses available Doses opened/used Ending balance (open, unopened vials)
Daily summary sheet, health facility	Doses administered by age and sex Number of vials opened
Logistics Management Information System (LMIS)	Ending balance Doses issued Doses received Doses available Doses consumed
Health Management Information System (HMIS)	Doses administered (by EPI schedule) Doses opened
National Statistics Institute (INE)	Population estimates by health facility

**Table 2:** Vaccines on the EPI schedule in Mozambique, their characteristics, and globally acceptable vaccine wastage rates.

Vaccine	Doses per Vial	Formulation	Multi-Dose Vaccine Policy (MDVP) Discard Time	Acceptable Vaccine Wastage Rates
BCG	20	Lyophilized	6 h	50%
MR	10	Lyophilized	6 h	25%
IPV	5	Liquid	28 days	15%
OPV	10	Liquid	28 days	10%
DPT (Pentavalent)	10	Liquid	28 days	15%
Rota	1	Liquid	n/a	5%
PCV	4	Liquid	28 days	5%

**Table 3:** EPI schedule and session cohorts based on timing of vaccination.

Cohort	Age for Vaccination	Planned Vaccines
Session Cohort 1	Birth	BCG
		OPV 0
Session Cohort 2	2 months	DPT 1
		OPV 1
		PCV 1
		RV 1
Session Cohort 3	3 months	DPT 2
		OPV 2
		PCV 2
		RV 2
Session Cohort 4	4 months	DPT 3
		OPV 3
		IPV
Session Cohort 5	9 months	PCV 3
		MR 1
Session Cohort 6	18 months	MR 2

**Table 4:** Results of data collection by sources and number of facilities.

Source	Nhamatanda	Gorongosa	Total
Stock card August–October 2023	21 facilities	15 facilities	36 facilities
Daily summary sheets August–October 2023	8 facilities	9 facilities	17 facilities
HMIS August–October 2023	22 facilities	20 facilities	42 facilities
HMIS January–December 2023	n/a	17 facilities	17 facilities
LMIS July–October 2023	n/a	16 facilities	16 facilities
INE target population estimates 2023	n/a	17 facilities	17 facilities

**Table 5:** Description of data collection from all sources, and quality summary.

	Nhamatanda	Gorongosa
Average number of stockouts among facility/vaccine combinations (defined by a reported zero balance at the end of the month in the LMIS)	n/a	158/576 (27%)
Match of consumption reported in the LMIS with open vials reported in the HMIS	n/a	354/480 (74%)
Among the mismatched figures, the proportion of LMIS consumption reported in the HMIS as opened (*n* = 28)	n/a	342% BCG 92% all others
Completeness of stock cards	64/176 (36%)	9/120 (8%)
Aggregate administered doses of BCG, DPT, IPV, MR, PCV, OPV, and Rota per HMIS	74,333 (59%)	51,014 (41%)

## Data Availability

Data are available upon request from the corresponding author, with all facility identifier information removed.
